# Electrical stimulation of biofidelic engineered muscle enhances myotube size, force, fatigue resistance, and induces a fast‐to‐slow‐phenotype shift

**DOI:** 10.14814/phy2.70051

**Published:** 2024-10-09

**Authors:** Isabella Pallotta, Michael J. Stec, Brian Schriver, David R. Golann, Kevin Considine, Qi Su, Victor Barahona, Julia E. Napolitano, Sarah Stanley, Meghan Garcia, Nicole T. Feric, Krista M. Durney, Roozbeh Aschar‐Sobbi, Nathan Bays, Tea Shavlakadze, Michael P. Graziano

**Affiliations:** ^1^ Valo Health Boston Massachusetts USA; ^2^ Regeneron Pharmaceuticals Tarrytown New York USA

**Keywords:** dexamethasone, electrical stimulation, engineered skeletal muscle, skeletal muscle contractility

## Abstract

Therapeutic development for skeletal muscle diseases is challenged by a lack of ex vivo models that recapitulate human muscle physiology. Here, we engineered 3D human skeletal muscle tissue in the Biowire II platform that could be maintained and electrically stimulated long‐term. Increasing differentiation time enhanced myotube formation, modulated myogenic gene expression, and increased twitch and tetanic forces. When we mimicked exercise training by applying chronic electrical stimulation, the “exercised” skeletal muscle tissues showed increased myotube size and a contractility profile, fatigue resistance, and gene expression changes comparable to in vivo models of exercise training. Additionally, tissues also responded with expected physiological changes to known pharmacological treatment. To our knowledge, this is the first evidence of a human engineered 3D skeletal muscle tissue that recapitulates in vivo models of exercise. By recapitulating key features of human skeletal muscle, we demonstrated that the Biowire II platform may be used by the pharmaceutical industry as a model for identifying and optimizing therapeutic drug candidates that modulate skeletal muscle function.

## Graphical abstract

Biofidelic engineered skeletal muscle tissues in the Biowire II platform.

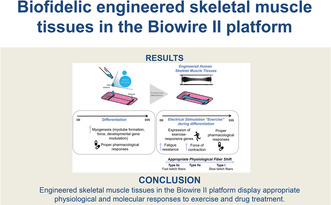

## 1 INTRODUCTION

Skeletal muscle dysfunction can be caused by a spectrum of genetic mutations (e.g., Duchenne muscular dystrophy) or in settings of aging, inflammation, cancer (cachexia), immobilization and several other conditions (Mukund & Subramaniam, 2020). More recently, clinical studies have also reported fatigue or muscle weakness as a long‐term symptom of post‐COVID‐19 infection (Huang et al., 2021), and muscle mass and strength have been shown to be predictors of length of hospital stay in patients with moderate to severe COVID‐19 (Gil et al., 2021). Regardless of the etiology, skeletal muscle disorders result in muscle atrophy, degeneration, or dysfunction, and impairment of the patient's quality of life. Unfortunately, to date, there are limited treatment options for many musculoskeletal and neuromuscular disorders, in part due to the limited predictive value/capability of the models currently available for therapeutic testing, specifically 2D tissue culture and preclinical animal models.

Major challenges of using animal models for skeletal muscle research are due to species differences that limit clinical translatability of findings (Hay et al., 2014; Lowe & Alway, 2002; Sztretye et al., 2020). Cultured 2D models of skeletal muscle are less labor‐intensive than animal models; however, their limitations include a difficulty to maintain cultures in the long‐term, resulting in cellular immaturity and morphology that is not consistent with mature innervated musculature, as well as a lack of a mature contractile response (Cooper et al., 2004; Guo et al., 2014). The inability to fully recapitulate the organization and function of mature skeletal muscle in 2D cultures inevitably limits the utility of these systems for identifying new insights in muscle pathophysiology and drug discovery.

This clinical need prompts the development of novel tissue culture methods that are better able to model the complex pathophysiology of human skeletal muscle and in turn advance the development of new treatments for muscle diseases. To this end, engineered 3D human skeletal muscle models represent a promising approach to overcome some of the limitations of 2D cell culture systems and better complement pre‐clinical studies (reviewed in (Broer et al., 2020; Khodabukus, 2021)). Engineered 3D models can recreate complex tissue architectures by providing structural and mechanical features that better resemble native tissues. By embedding cells in hydrogel, wherein cells can tri‐dimensionally anchor, cell‐extracellular matrix (ECM) interactions are formed that are critical for outside‐in biochemical signaling (Yamada et al., 2022). In vivo, the ECM not only provides a structural scaffold for skeletal myofiber contractility, but also plays a critical role in molecular signaling. Thus, by modulating the hydrogel composition and stiffness, environments that recapitulate healthy or pathological conditions can be generated. Additionally, it has been demonstrated that embedding myoblasts in a hydrogel anchored between two attachment points can induce myotube alignment by recreating mechanical features present in the native skeletal muscle, and mechanical tension and alignment can promote sarcomere maturation (reviewed in (Jalal et al., 2021)). Perhaps more importantly, these advantages of engineered 3D skeletal muscle tissue models can promote a physiologically relevant contractile phenotype. In the last decade, a variety of 3D skeletal muscle models have been proposed (reviewed (Cho & Jang, 2021; Jalal et al., 2021)). However, novel platforms are needed to expand this burgeoning field in order to advance towards models progressively closer to the complexity of human physiology. For instance, an important physiological feature that has not been achieved yet in human 3D skeletal models is muscle fatigue resistance compatible with physiology. The ability to recapitulate physiological fatigability in a tissue culture setting would enable investigation into the mechanism of a wide range of disease states and accelerate drug screening.

We recently reported the use of the Biowire II platform for generating functional 3D engineered cardiac tissues that display several hallmarks of the adult human myocardium, as well as expected responses to known compounds (Feric et al., 2019; Zhao et al., 2019). Both cardiac and skeletal muscle have been demonstrated to require electromechanical stimulations to mature. The Biowire II platform consists of two elastic polymer wires between which skeletal muscle tissues can be suspended, recreating the mechanical tension typical of the native skeletal muscle environment. Additionally, the Biowire II platform offers the ability to apply external electrical stimulation to further promote tissue maturation. In the present study, we aimed to adapt the Biowire II platform to engineer a physiologically relevant 3D model of human skeletal muscle tissue. The combination of the platform and skeletal muscle‐specific culture conditions allowed for differentiation of primary human myoblasts into myotubes and increased twitch and tetanic force during long‐term culture. Seven days after the myoblasts were seeded in the Biowire II platform, electrical field stimulation designed to mimic skeletal muscle exercise training was applied to a subset of the tissues. The electrical stimulation improved myotube size and force production, showing the highest tetanic/twitch ratio as compared to what has been reported to date in 3D skeletal muscle models. Importantly, here we show that “exercise” (electrical stimulation) improved fatigue resistance in a human model, and that exercise induced a shift in gene expression from fast myosin heavy chain (MyHC) isoforms to slower MyHC isoforms, a shift commonly reported in vivo with endurance exercise training. Additionally, the 3D skeletal muscle tissues responded as expected to a well characterized catabolic steroid, dexamethasone, in terms of myofiber contractility and gene expression changes. Altogether, these results validate an engineered 3D human skeletal muscle model that recapitulates important hallmarks of human muscle physiology and that can be used in drug discovery and disease modeling applications.

## 2 MATERIALS AND METHODS

### 2.1 Engineered human skeletal muscle tissue formation

Primary human skeletal muscle cells (skMDC) (Cook Myosite, Pittsburgh, PA) and dermal fibroblasts (Lonza, Allendale, NJ, CC‐2509) were maintained in 2D culture following the manufacturer's recommendations. Specifically, skMDCs and dermal fibroblasts were maintained in the following growth media: Cook Myosite, Pittsburgh, PA, Myotonic Basal Medium MB‐2222‐0059 and Myotonic Growth Supplement MS‐3333‐029, for skMDCs; Lonza, Allendale, NJ, CC‐3132, for dermal fibroblasts. For skeletal muscle tissue formation, first a hydrogel was prepared by combining fibrinogen (Sigma Aldrich, Burlington, MA, F3879) (final concentration 5 mg/mL) with 20% (v/v) Matrigel (VWR, Bridgeport, NJ, 356230) and 4% (v/v) 25 U/mL thrombin (Sigma Aldrich, Burlington, MA, T6884). Dissociated skMDCs and dermal fibroblasts were mixed in a 20:1 (skMDC: fibroblasts) cell number ratio, pelleted and resuspended at a concentration of 4.73 × 10^7^ cells/mL in a hydrogel. This results in a 90,000 skMDCs and 4500 dermal fibroblasts per tissue on average. The cell‐hydrogel suspension (2 μL per well) was seeded into the Biowire II platform, consisting of 5 mm long × 1 mm wide x 0.3 mm deep polystyrene microwells containing parallel poly (octamethylene maleate (anhydride) citrate) (POMaC) wires (Feric et al., 2019). Tissues were cultured for 2 days in growth media (Cook Myosite, Pittsburgh, PA, MB‐2222‐0059 and MS‐3333‐029) and subsequently cultured in NbActiv4 differentiation media (Transnetyx Tissue, Cordova, TN, NB4). Media was changed twice per week. At day 7 of differentiation, some tissues were subjected to intermittent external electrical stimulation (in parallel) in custom chambers containing parallel carbon electrodes (Feric et al., 2019) for 1 h twice per day (10 Hz every 10 s, 4 ms pulse duration).

In some experiments, skMDCs were maintained in 2D culture for the entire differentiation process. Briefly, skMDCs (Cook Myosite, Pittsburgh, PA) were maintained in 2D culture by following the manufacturer's recommendations. Once reaching about 90% confluency, cells were switched to NbActiv4 differentiation media. NbActiv4 differentiation media was changed twice per week.

### 2.2 Immunofluorescence analysis of engineered human skeletal tissues

For super‐resolution microscopy, skeletal muscle tissues were fixed in paraformaldehyde for 30 min and subsequently permeabilized with 0.1% Triton for an additional 30 min at room temperature (RT). After washing with PBS, tissues were incubated with Alexa Fluor 488 Phalloidin (ThermoFisher Scientific, Waltham, MA, A12379), diluted 1:400 in PBS, for 1 h at RT. After washing with PBS, nuclei were counterstained with DAPI. Images were acquired using a CSU‐W1 spinning disk confocal and analyzed using NIS‐Elements software (Nikon Instruments Inc, Edgewood, New York). Super resolution images were acquired using a CSU‐W1 SoRa spinning disk confocal system (Nikon Instruments Inc, Edgewood, New York). In some experiments, tissues were washed in PBS with 10 mM KCl for 5 min prior to fixation, to prevent tissue contraction. After permeabilizing, tissues were stained with alpha‐actinin (Sigma Aldrich, A7811) diluted 1:800 in PBS, for 1 h at RT, followed by an Alexa Fluor 488 conjugated secondary antibody (ThermoFisher Scientific, Waltham, MA, A‐21202) diluted 1:500 for 1 h at RT. Sarcomere length was extrapolated by using NIS Elements software.

For F‐Actin staining for myotube diameter quantification, tissues were fixed in paraformaldehyde for 30 min and washed with PBS. After three 5‐min PBS washes, tissues were permeabilized in blocking solution (20% goat serum, 0.3% Triton X‐100 in 1× PBS) for 1 h at RT. Tissues were incubated with fluorophore‐conjugated phalloidin (1:500; Invitrogen, A22287) in blocking solution overnight at 4°C. Tissues were then washed three times for 5 min in PBS and then mounted in Fluoromount‐G (ThermoFisher, 00–4958‐02). Images were acquired using a LSM 710 Confocal (Zeiss) and processed in Zen blue edition 2.0. Average maximal myotube diameter was quantified using HALO software (Indica Labs) by tracing the maximal diameter of 53–111 myotubes per tissue, and the average maximal diameter of all myotubes within a tissue was reported.

### 2.3 Gene expression analysis of engineered human skeletal muscle tissues

Tissues were flash frozen in liquid nitrogen and stored at −80°C until processing. Tissues were homogenized in Trizol and total RNA was purified using MagMAX™‐96 for Microarrays Total RNA Isolation Kit (Ambion by Life Technologies, AM1839) according to manufacturer's specifications. Genomic DNA was removed using MagMAX™Turbo™DNase Buffer and TURBO DNase from the MagMAX kit listed above (Ambion by Life Technologies). mRNA (up to 2.5ug) was reverse‐transcribed into cDNA using SuperScript® VILO™ Master Mix (Invitrogen by Life Technologies, 11,755,500). cDNA was diluted to 0.5‐5 ng/uL. 2.5‐25 ng cDNA input was amplified with the SensiFAST Hi‐ROX MasterMix (1 × 100 mL) (BIOLINE, CSA‐01113) using the ABI 7900HT Sequence Detection System (Applied Biosystems). The following TaqMan assays were used from Thermo Scientific: MYH1 (Hs00428600_m1), MYH2 (Hs00430042_m1), MYH3 (Hs01074199_g1), Myh7 (Hs01110632_m1), MYH8 (Hs00933054_m1), MYOG, Fwd‐5′‐TGCCCACAACCTGCACTC, Rev‐5′‐GGAAGGCCACAGACACATC, probe‐5′‐CCTCACCTCCATCGTGGACAGCA.

For RNAseq analysis, tissues were processed using the Qiagen miRNAeasy micro kit (Qiagen, 1,071,023) and stored at −80°C until further processing. mRNA‐seq libraries were generated using KAPA® mRNA HyperPrep Kit (Roche Sequencing). Starting material was 10 ng RNA and fragmentation was done at 85°C for 6 min. cDNA was ligated with 300 nM xGen Dual Index UMI Adapters (Integrated DNA Technologies) and amplified using 16 PCR cycles. Sequencing of the resulting libraries was done on NovaSeq 6000 (Illumina) using a 51 cycle, single‐end sequencing recipe. Raw sequence data (BCL files) were converted to FASTQ format via bcl2fastq v2.20. Reads were mapped to the human transcriptome (NCBI GRCh37) using ArrayStudio® software (OmicSoft®, Cary, NC) allowing zero mismatches. Reads mapped to the exons of a gene were summed at the gene level.

DESeq2 (1.34.0) used a pair‐wise design to measure differential expression and significantly perturbed genes were defined with fold changes no less than 1.5 in either up or down directions with Benjamini‐Hochberg adjusted p value of <0.05. Variance Stabilizing Transformation (VST) was performed to remove effects due to difference between samples. Genes with less than 10 reads in 80% of samples in the higher expressed group were excluded from the final significantly perturbed gene list. For these differentially expressed genes, we performed pathway enrichment analysis using BaseSpace's correlation engine (basespace.illumina.com) to investigate biological functions. Both RT‐qPCR and RNAseq analysis were performed on individual tissue samples.

### 2.4 Contractility measurements of engineered human skeletal tissues

**FIGURE 1:**
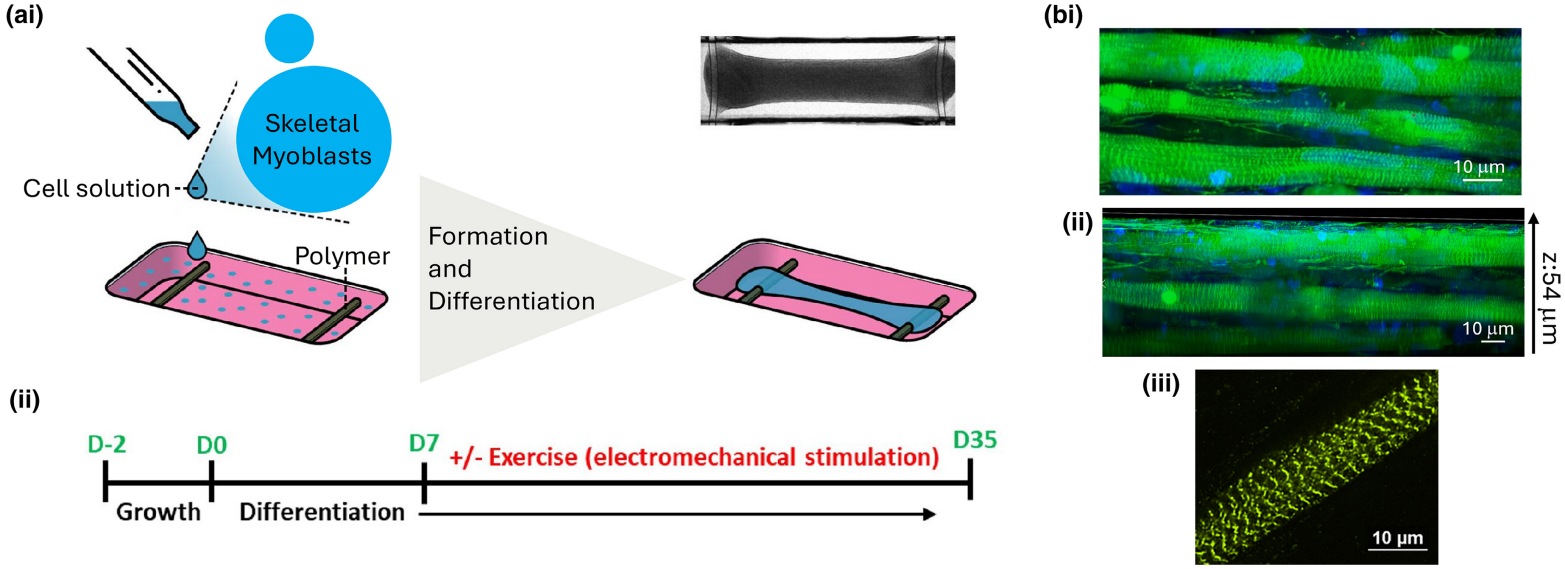
Engineering 3D human skeletal muscle tissues in the Biowire II platform. (a) Schematic representation of 3D skeletal muscle tissue formation. Cell (myoblasts and fibroblasts) suspension in a hydrogel is seeded in the Biowire II platform. Tissue compaction occurs over time, resulting in tissues suspended between 2 polymer wires (i). Seeded cells were maintained for 2 days in growth media and then switched to differentiation media for 5 weeks. On day 7 of differentiation, a subset of skeletal muscle tissues was exercised by electrical field stimulation (ii). (b) Representative immunofluorescent images of skeletal muscle tissues composed of myotubes stained with fluorophore‐conjugated phalloidin to detect F‐Actin (green) and nuclei stained with DAPI (blue) (i), and myotube distribution in a 54 μm z stack (ii). Representative immunofluorescence image of a myotube, stained with alpha actinin, showing a clear sarcomere structure (iii).

Skeletal muscle tissue contractility was measured by tracking the deflection of the POMaC wires as a function of time, as previously described (Feric et al., 2019). Tissues were transferred to a custom chamber containing parallel carbon electrodes to provide external field stimulation in an environmental chamber at 37°C and 5% CO2. The wires were illuminated using 350 nm excitation. Videos were acquired using a Zyla 4.2 sCMOS camera (Andor, South Windsor, CT) with a 470 nm emission filter using NIS‐Elements software. To measure contractile force, the videos were analyzed using a custom Matlab program. Twitch and different degrees of tetanus were acquired by stimulating the tissues at 1 Hz, 5 Hz, 10 Hz, 20 Hz and 100 Hz for 6 s, 0.56 V/mm, 4 ms pulse duration. In some experiments, contractility was normalized to the cross‐sectional area of the muscle tissues. To determine the tissue cross‐sectional area, both unstimulated and stimulated tissues, at different maturation periods (3 tissues/group) were fixed in 10% neutral buffered formalin for 30 min, followed by 3 washes in PBS. After embedding in paraffin, tissues were cut at 3 locations (red dotted lines in Figure 1aiii) and 4 sections/location were obtained. An image of each transverse section was acquired, and the median cross‐sectional area was determined using Aperio ImageScope[v12.4.6.5003], from Leica Biosystems. Fatigability was measured as loss of force generation during a 10 min‐repeat submaximal tetanic stimulus (80 Hz, 0.4 s duration, 0.4 s rest between submaximal tetanic stimuli) that resulted in 50% force reduction from time zero.

### 2.5 Drug testing of engineered human skeletal muscle tissues

Two week‐stimulated tissues and time‐matched unstimulated tissues were treated with 10 **μ**M dexamethasone (Sigma Aldrich, St. Louis, MO, D4902) or vehicle control. 1000× dexamethasone stock solution was prepared in ethanol. Compounds and vehicle controls, in experimental and control tissues respectively, were replaced every 2 days during full media change for 14 days. Tissues were analyzed weekly for contractility, as described above. Each tissue's contractility was first normalized to relative baseline, and then subsequently to vehicle controls.

### 2.6 Statistics

Analysis by one‐way or two‐way ANOVA was followed by Bonferroni's post‐hoc testing. Student's t‐test was performed for paired observations. A value of *p* < 0.05 was considered statistically significant. Statistical analysis was performed using GraphPad Prism 8.0 software. All experiments were independently replicated at least three times unless otherwise noted.

## 3 RESULTS

### 3.1 Engineering 3D human skeletal muscle tissues

Human skeletal muscle tissues were generated by encapsulating primary human skeletal muscle cells in a hydrogel composed of Matrigel and fibrin, and then seeding the cell suspension into the Biowire II platform, as described in Materials and Methods. The efficiency of our platform, in terms of tissue yield, that is successful tissue generation, was approximately 70%. To mimic skeletal muscle physiology, fibroblasts were added in a 1:20 ratio to both provide structural support through ECM protein synthesis (Chapman et al., 2016), and stimulate myogenesis (Mackey et al., 2017). Moreover, fibroblasts are able to exert contractile forces on the hydrogel and thereby facilitate compaction via fibroblast extension and interaction with the hydrogel through integrin binding (Hinz et al., 2001; Tamariz & Grinnell, 2002). Additionally, softer hydrogels, such as those composed of Matrigel and fibrin, have been demonstrated to improve the contractility of engineered 3D skeletal models relative to stiffer milieu (Hinds et al., 2011). In our experimental conditions, myoblasts and fibroblasts self‐organized and gradually remodeled the hydrogel to form a tissue suspended between two polymer wires after 24 h (Figure 1ai). Skeletal muscle tissues were cultured for 2 days in the platform before differentiation was initiated and then monitored for another 35 days. On Day 7 of differentiation, the skeletal muscle tissues were divided into groups, wherein one group was subjected to an exercise regime, consisting of intermittent external electrical stimulation and the other group was maintained as an unstimulated control culture (Figure 1aii).

Confocal microscopy analysis of skeletal muscle tissues immunostained with F‐actin demonstrated that at the end of the maturation protocol, tissues were composed of fused multinucleated myotubes (Figure 1bi) and these myotubes were tri‐dimensionally distributed (Figure 1bii, Movie S1). One defining property of skeletal muscle in vivo is the presence of sarcomeres, which are responsible for force production. Super‐resolution images at 240x magnification, acquired using a SoRa spinning disk confocal system, allowed for visualization and quantification of sarcomeres in the myotubes (Movie S2). The sarcomere length, measured as the distance between Z‐lines, was 2.36 ± 0.15 μm (Figure 1biii), falling within the range of the human myofiber sarcomere length (Cutts, 1988).

### 3.2 Effect of differentiation time on engineered 3D human skeletal muscle tissue structure and function

**FIGURE 2:**
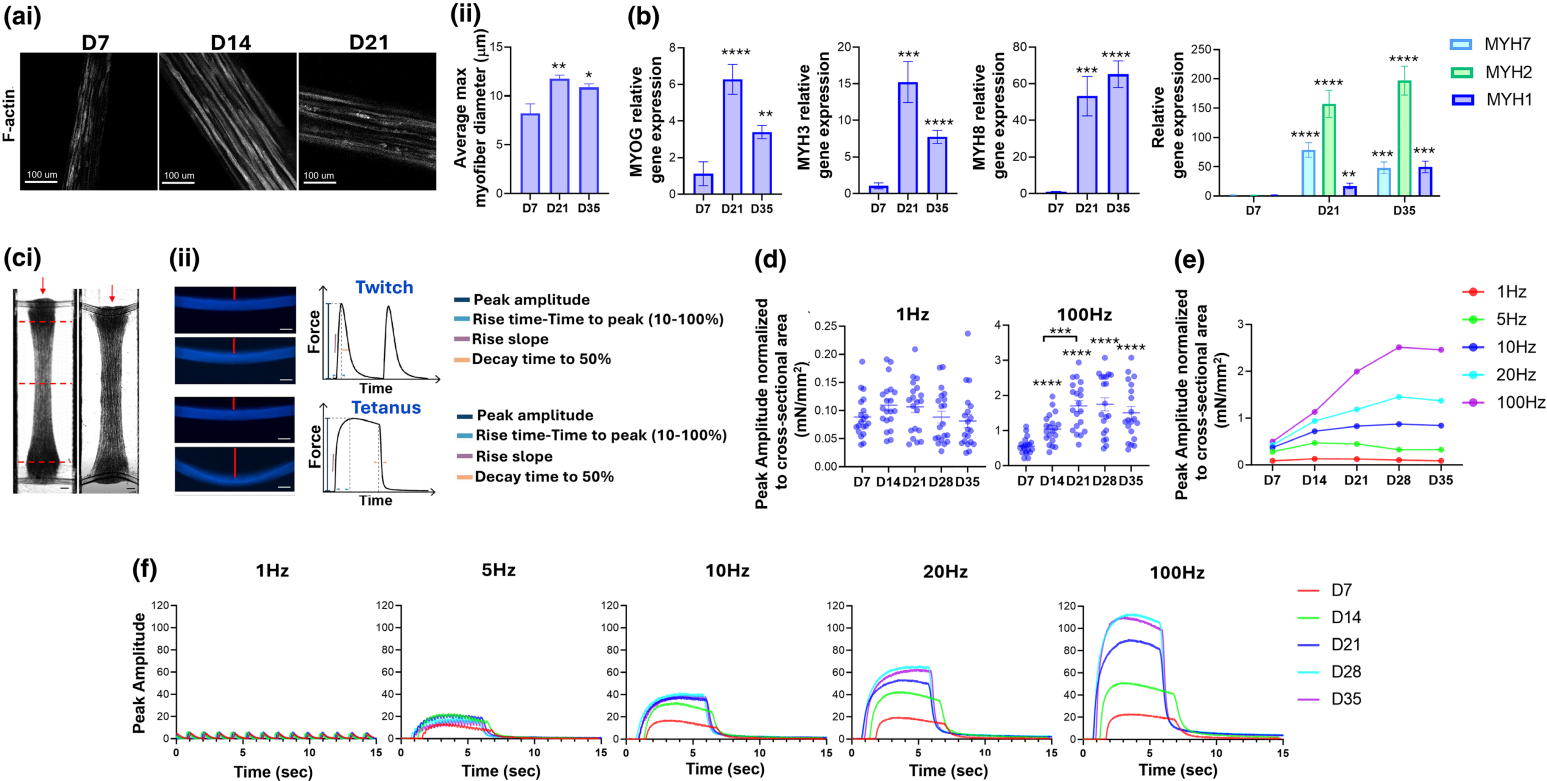
Increasing culturing time in differentiation media enhances myotube formation and modulates developmental gene expression and contractility. (a) Confocal microscopy images of skeletal muscle tissues immunostained with fluorophore‐conjugated phalloidin to detect F‐Actin (i) and relative quantification of myotube diameter (ii), showing increasing myotube size over maturation period. (b) Myogenic/developmental gene expression over maturation time. Bars represent mean ± SEM (*n* = 20). (c) Contractile forces are measured non‐invasively by optically tracking the deflection of the polymer wire under electrical field stimulation (i, ii). (d) Skeletal muscle tissues generate tetanic forces starting at day 7 of differentiation, and tetanic forces in response to 100 Hz electrical stimulation increase over maturation time. Data shown as individual tissue values, line represents mean ± SEM (*n* = 20). (e, f) Representative traces of skeletal muscle tissues showing increasing forces at increasing electrical stimulation frequencies and over increasing maturation periods. **p < 0.005.

Skeletal muscle tissues were analyzed structurally over time while in culture in a differentiation media. F‐actin staining revealed myotubes were well aligned and oriented with respect to axis of actuation and enhanced myotube formation occurred over time (Figure 2ai). Quantification of myotube diameter resulted in a statistical increase in diameter at day 21 as compared to day 7 of differentiation (8.22 ± 0.90 **μ**m at day 7; 11.76 ± 0.3 **μ**m at day 21). At day 35, we did not observe a further increase in myotube diameter relative to day 21 (Figure 2aii). Additionally, the combination of the Biowire II platform and differentiation time modulated developmental gene expression. For example, mRNA expression of myogenin (MYOG) and embryonic (MYH3) MyHC increased from day 7 to 21, however declined from day 21 to day 35 (Figure 2b). Neonatal MyHC (MYH8) mRNA expression increased as a function of differentiation time (Figure 2b). Interestingly, at day 21 and day 35 of differentiation, engineered skeletal muscle tissues expressed adult MyHC isoforms (MYH1, MYH2, MYH7) (Figure 2b), pointing to a more mature phenotype of the cultures. These adult isoforms were not detected at day 7 following differentiation (Figure 2b).

In contrast to 3D cultured skeletal muscle tissues, when cells were differentiated in 2D, we could only maintain the cultures for at most 11 days before cell detachment occurred. On day 10 of the 2D differentiation, we observed increased mRNA expression of *MYOG*, *MYH1* and *MYH2*, as compared to day 0 of differentiation (Figure S1ai). However, when compared to unstimulated tissues (Day 21), the 2D skeletal muscle cells (Day 10) expressed significantly less *MYH1* and *MYH2* (Figure S1aii). Additionally, we observed less myotube formation in 2D skeletal muscle cells compared to unstimulated tissues, and the 2D myotubes that formed were randomly orientated (Figure S1bi,ii).

The Biowire II platform allows for measuring parameters of contractility by imaging the deflection of a polymer wire as a function of time (Figure 2 ci–ii, as previously reported (Feric et al., 2019). Specifically, we can extrapolate the amplitude (force), rise time (time‐to‐peak), rise slope (contraction rate) and decay time to 50% (half‐time relaxation) of 1 Hz twitches and tetanus generated using high frequencies (Figure 2cii). At 7 days of differentiation, skeletal muscle tissues generated tetanic forces with high frequency stimulation (at 100 Hz, 0.55 ± 0.23 mN/mm^2^; at 1 Hz, 0.09 ± 0.03 mN/mm^2^) (Figure 2d). The force at 1 Hz field stimulation did not change over 35 days of differentiation; however, the tetanic force generated by human muscle cultures at 100 Hz stimulation increased from day 7 to day 21 and plateaued thereafter to day 35 of differentiation (Figure 2d). Similar to 100 Hz, skeletal muscle tissues showed a time‐dependent increase in force at 5 Hz, 10 Hz and 20 Hz, as shown in the representative traces in Figure 2e,f.

### 3.3 Exercise modulates skeletal muscle tissue morphology, gene expression, contractility, and fatigability

**FIGURE 3:**
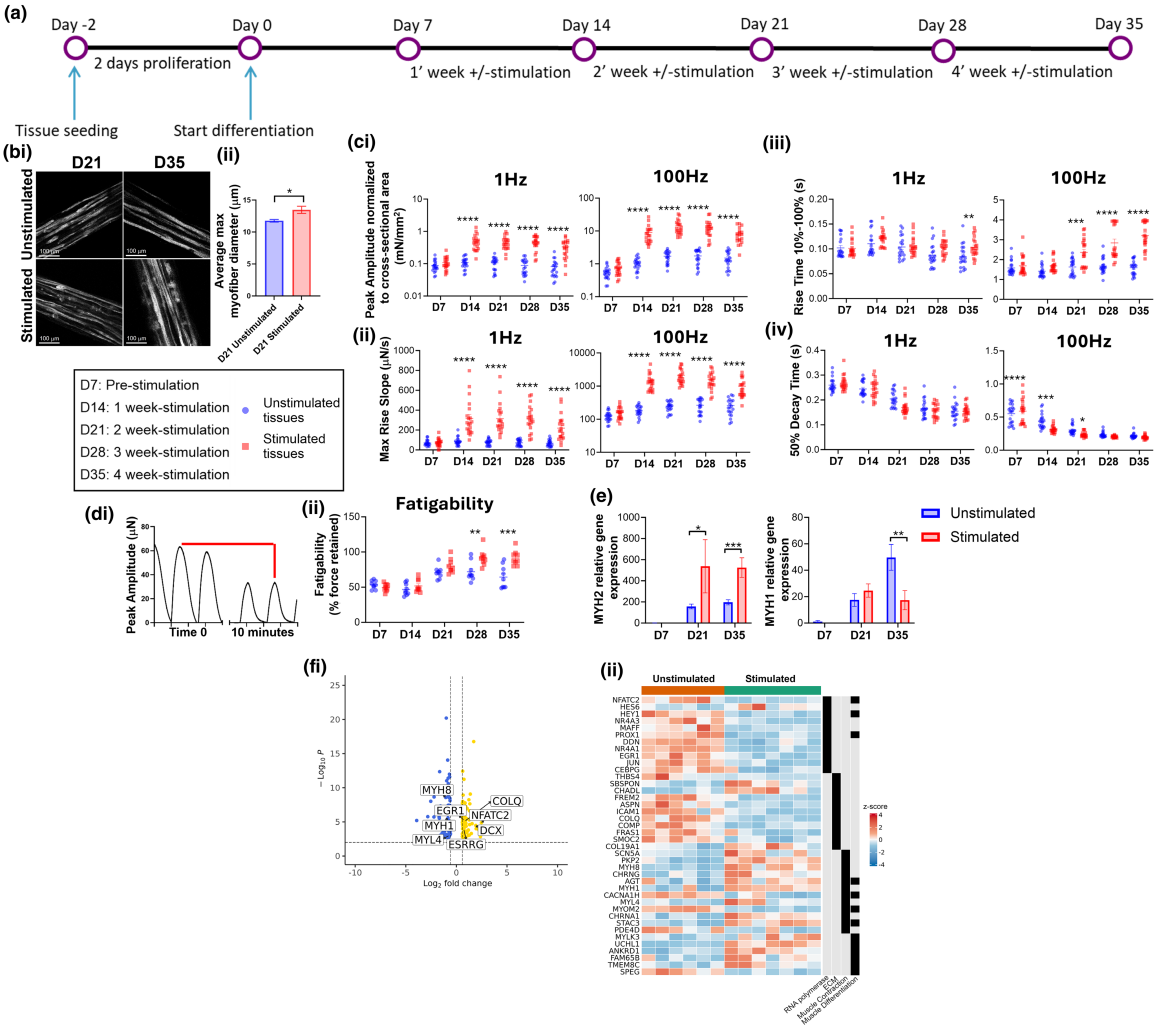
Exercise enhances myotube formation and force production/fatigability in association with exercise‐related gene expression changes. (a) Experimental design schematic. (b) Confocal analysis of skeletal muscle tissues immunostained with fluorophore‐conjugated phalloidin to detect F‐Actin (i) and relative quantification of myotube diameter (ii), showing increased myotube size in D21 stimulated tissues compared to time‐matched unstimulated tissues. Bar represents mean ± SEM. (c) Stimulated tissues show increased peak amplitude (force) (i), rise slope (contraction rate) (ii), rise time (time‐to‐peak) (iii) at both 1 Hz and 100 Hz, and reduced decay time (half‐time relaxation) at 100 Hz over time (iv). Data shown as individual tissue values, line represents mean ± SEM (*n* = 20). (d) Fatigability is measured as 50% force reduction during a 10 min‐repeat submaximal tetanic stimulus (i). Exercise results in tissues with increased fatigue resistance over maturation periods (ii). Data shown as individual tissue values, line represents mean ± SEM (*n* = 9). Statistics have been computed by two‐way ANOVA, followed by Bonferroni's post hoc test. (e) Exercise results in the modulation of adult MyHC expression, specifically a IIx to IIa MyHC shift. Bar represents mean ± SEM. (f) Volcano plot of RNAseq data depicting genes that were significantly upregulated by exercise (e.g., ESRRG, NFATC2), as well as developmental and fatigable contractile genes (e.g., MYH8, MYH1) that were downregulated by exercise (i). Gene ontology analysis identified pathways involved in RNA polymerase, ECM, and Muscle Contraction/Differentiation to be differentially regulated with stimulation, with expression of select genes from these pathways depicted in heatmap format, with z‐score normalization of gene counts representing expression between tissues (*n* = 6–7) (ii). *p < 0.05; **p < 0.005; ***p < 0.0005; ****p < 0.0001.

At day 7 of tissue differentiation, we applied a 4‐week intermittent electrical stimulation protocol (Figure 3a), designed to mimic muscle exercise training. F‐actin immunostaining demonstrated that this exercise protocol enhanced myotube formation (Figure 3bi). Specifically at day 21 of differentiation (corresponds to 2‐weeks stimulation), myotube diameter was larger in electrically stimulated tissues, compared with unstimulated tissues (Figure 3bii). We also observed enhanced contractility with exercise. Specifically, after 1 week of stimulation (day 14 of differentiation), exercised skeletal muscle tissues displayed a significant increase in force production and rise slope (contraction rate) both at 1 Hz and 100 Hz stimulation relative to unstimulated tissues. Although the contraction force produced by stimulated tissues reached a plateau after 2 weeks of stimulation and subsequently decreased, the force generated by stimulated tissues was significantly higher compared with unstimulated tissues at all time points (Figure 3 ci, ii, Figure S2). Rise time (time‐to‐peak) was similarly increased by exercise, however only at the later time points, starting from day 21 of differentiation at 100 Hz and day 35 of differentiation at 1 Hz (Figure 3ciii). Finally, when stimulated at 100 Hz, decay (half‐time relaxation) time of exercised skeletal muscle tissues was significantly lower compared to unstimulated tissues (Figure 3civ). Altogether, these data demonstrate that the electrical stimulation regime applied to the tissues resulted in dramatic modulations of their contractility parameters.

Skeletal muscle fatigability is the loss of force in response to prolonged contractile activity, which occurs both physiologically and in diseased states. It is well‐known that exercise training can enhance muscle fatigue resistance through multiple mechanisms (Egan & Zierath, 2013). To investigate if our exercise regime was able to enhance muscle fatigue resistance, we developed a fatiguing protocol consisting of 10 min of repeated submaximal tetanic stimuli, which resulted in a maximal force reduction of ~50% at day 7 of differentiation. We then measured fatigability weekly in stimulated and unstimulated tissues and found that with chronic electrical stimulation (exercise), fatigue resistance of skeletal muscle tissues improved by 20% and 27% at week 3 and 4, respectively, compared to non‐exercised tissues (Figure 3d).

To investigate the molecular changes underlying improved contractile performance and fatigue resistance evoked by exercise training, MyHC isoform gene expression was analyzed. Interestingly, unstimulated tissues expressed more Type IIx MyHC (MYH1) than stimulated tissues, while exercised tissues expressed more Type IIa MyHC (MYH2) than unstimulated tissues (Figure 3e). The shift from the more fatigable IIx to the more fatigue resistant IIa MyHC isoform is a commonly observed adaptation to human exercise training (Short et al., 2005), demonstrating that long‐term stimulation in the Biowire II platform recapitulates some aspects of in vivo exercise training. To further examine the molecular response of engineered skeletal muscle to exercise, we performed RNAseq analysis on unstimulated and stimulated tissues. Compared to unstimulated tissues, stimulated tissues had increased expression of 78 genes and decreased expression of 111 genes (Figure 3f). Gene ontology pathway analysis revealed several pathways that were differentially regulated between groups, including: RNA polymerase, ECM, Muscle Contraction, and Muscle Differentiation (Figure 3fii). Among the differentially expressed genes, several genes that are known to be induced following exercise or growth/regeneration stimuli were upregulated in stimulated tissues, including ESRRG, NFATC2, EGR1, DCX, and COLQ (Horsley et al., 2001; Irrcher & Hood, 2004; Lau et al., 2008; Ogawa et al., 2015; Rangwala et al., 2010). Additionally, downregulation of embryonic/fetal myosin heavy and light chain genes, MYH8 and MYL4, suggests increased maturity of stimulated tissues, and downregulation of MYH1 (IIx myosin heavy chain) in stimulated tissues supports qPCR gene expression findings of a fast IIx to fast IIa myofiber type shift in these tissues with exercise. While slow type I MyHC (MYH7) was expressed starting from D21, we did not observe any statistical difference between unstimulated and stimulated tissues (Figure S3). Finally, downregulation of acetylcholine receptor subunits α1 and γ (CHRNA1 and CHRNG, respectively) suggest that chronic stimulation may have modulated the post‐synaptic neuromuscular junction composition/morphology within the myotubes of these 3D tissues. Altogether, these data demonstrate that chronic electrical stimulation of engineered 3D human skeletal muscle tissues can mimic exercise training by enhancing tissue morphology and contractility, and that these changes are associated with induction or suppression of certain genes that are regulated in the same way by exercise in vivo.

### 3.4 3D human skeletal muscle tissues respond to chronic dexamethasone treatment by reduced contractility and induction of an atrophy program

**FIGURE 4:**
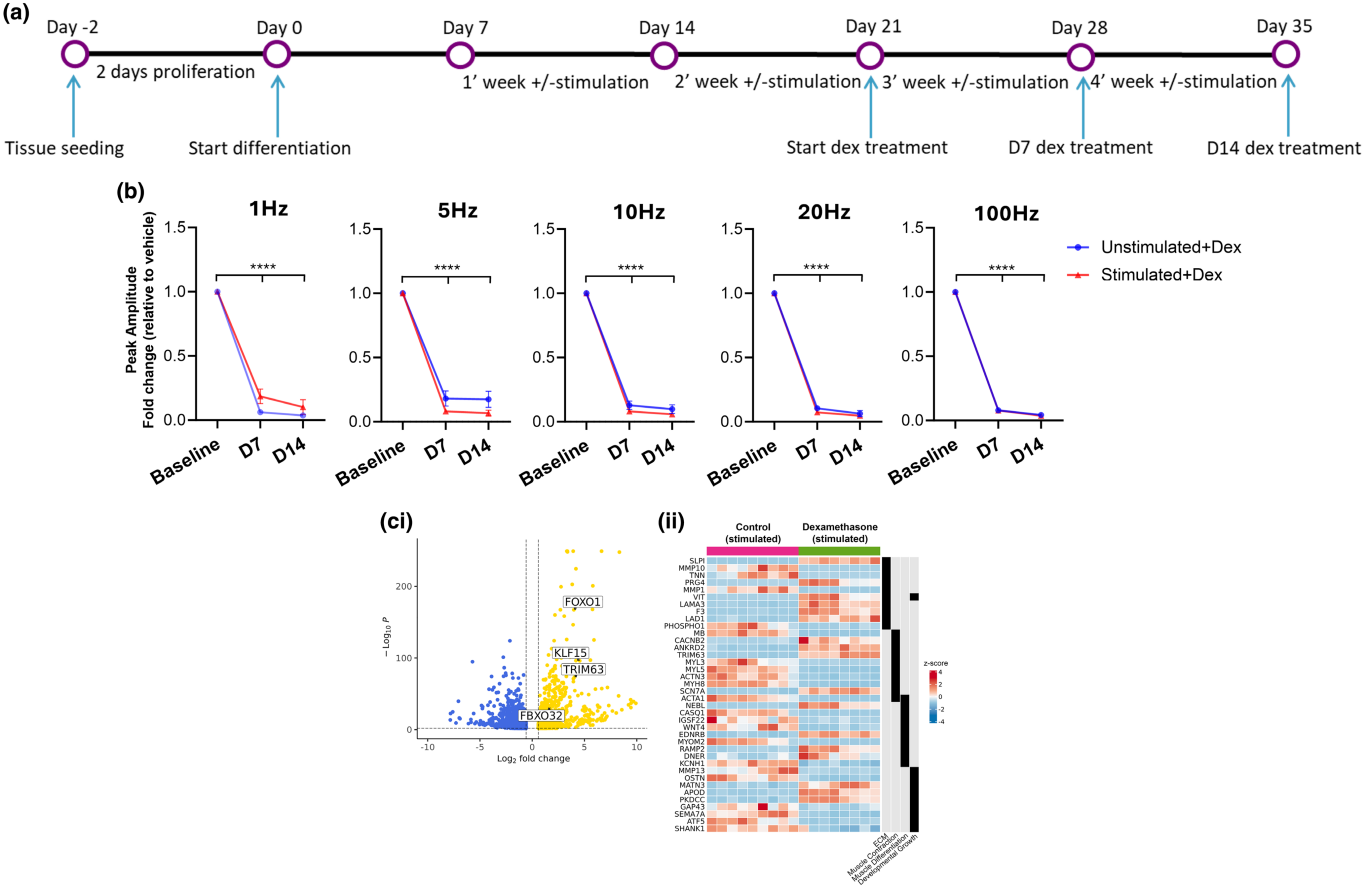
Dexamethasone treatment impairs force production in engineered human skeletal tissues and induces glucocorticoid‐responsive gene expression. (a) Experimental design diagram. (b) Chronic dexamethasone treatment decreases forces produced by both stimulated and unstimulated tissues. Data represent mean ± SEM (*n* = 8–9) *p < 0.05; **p < 0.005; ***p < 0.0005; ****p < 0.0001. (c) Volcano plot of RNAseq data depicting several genes that are known to mediate the response of muscle to glucocorticoid treatment, including KLF15, FOXO1, TRIM63, and FBXO32 (i). Gene ontology analysis identified pathways involved in ECM, Muscle Contraction/Differentiation, and Developmental Growth are altered by dexamethasone treatment, with expression of select genes from these pathways depicted in heatmap format, with z‐score normalization of gene counts representing expression between tissues (ii).

We investigated the response of our engineered skeletal muscle tissues to dexamethasone (Figure 4a), since the mechanism of action of this glucocorticoid has been well characterized in skeletal muscles. Synthetic glucocorticoids are widely used as anti‐inflammatory agents to treat musculoskeletal diseases, however a major side effect of such treatment is muscle atrophy. Dexamethasone directly induces skeletal muscle wasting by inducing FOXO‐mediated transcription of atrophy related genes, such as MuRF1/TRIM63 and Fboxo32/MAFbx, commonly known as atrogenes (Bodine et al., 2001; Clarke et al., 2007; Stitt et al., 2004) (reviewed in (Egerman & Glass, 2014)).

A one‐week treatment with dexamethasone dramatically decreased the force produced by both stimulated and unstimulated tissues in the Biowire II platform, and after 2 weeks of dexamethasone treatment, the force at all frequencies was further decreased (Figure 4b). We observed a similar large decrease in contraction and relaxation rate, as these parameters are a function of amplitude (data not shown). Dexamethasone treatment for 1 week also resulted in a significant increase in decay time (half‐time relaxation) in stimulated tissues, while not affecting rise time (time‐to‐peak) (Figure S4ai, ii); similar to what has been observed for mouse skeletal muscle treated with dexamethasone (Wang et al., 2023); however, this was not observed after 2 weeks of dexamethasone treatment (Figure S4Ai). To determine the molecular mechanisms responsible for the drastic decline in force production following dexamethasone treatment, RNAseq analysis was performed on stimulated tissues that were treated with either dexamethasone or a vehicle control for 14 days. Tissues treated with dexamethasone upregulated the expression of 1308 genes and downregulated the expression of 1504 genes compared to tissues that were treated with vehicle only (Figure 4c). Gene ontology pathway analysis revealed ECM, Muscle Contraction, Muscle Differentiation, and Developmental Growth to be differentially regulated between dexamethasone treated and vehicle treated groups (Figure 4cii). Activation of the glucocorticoid receptor by dexamethasone is known to induce expression of KLF15, and dexamethasone treated skeletal muscle tissues displayed a ~ 23‐fold increase in KLF15 expression compared to vehicle treated tissues. Additionally, glucocorticoids such as dexamethasone promote muscle atrophy via upregulation and nuclear translocation of FOXO transcription factors that induce atrogene expression (Clarke et al., 2007; Schakman et al., 2013; Stitt et al., 2004). Consistent with this, dexamethasone treated tissues significantly upregulated expression of FOXO1 and the atrogenes FBXO32 and TRIM63 (MAFbx and MuRF1, respectively) compared to vehicle treated tissues (Figure 4 ci). Overall, these data demonstrate the utility of our 3D human skeletal muscle platform for testing the effects of pharmacological drugs on muscle function and exploring molecular changes in response to drug treatment.

## 4 DISCUSSION

The pathophysiology of many skeletal muscle diseases is not fully understood, thus limiting therapeutic treatments. This clinical problem is mainly due to limited predictive models currently available, specifically traditional 2D in vitro and animal models. Here we engineered 3D in vitro human skeletal muscle tissues in the recently reported Biowire II platform (Feric et al., 2019).

To develop a functional 3D human skeletal muscle platform, we first optimized a tissue engineering protocol that allowed for the fusion of human myoblasts into myotubes in a defined 3D matrix. Myogenesis involves a sequence of coordinated events that include stem and progenitor cell proliferation, commitment, differentiation, and fusion to give rise to multinucleated skeletal myofibers (Bentzinger et al., 2012). These events are strictly controlled by the expression of developmental genes. For example, myogenin is a transcription factor that regulates terminal differentiation of myogenic progenitors during embryonic development (Andres & Walsh, 1996). Developing mammalian skeletal muscle is also characterized by the expression of unique contractile genes, including embryonic and neonatal myosin heavy chains, which are transiently expressed during development. After birth, developmental myosin isoforms are down‐regulated and are replaced with adult myosin isoforms (fast and slow myosins) (Schiaffino et al., 2015). In our engineered skeletal muscle tissues, culturing muscle cells in differentiation media from day 7 to day 21 promoted the expression of myogenin and embryonic/neonatal myosin heavy chains. By day 35 of differentiation, we were able to detect decreased expression of myogenin and embryonic myosin heavy chain, along with increased expression of adult myosin heavy chain genes (MYH7, MYH2 and MYH1). Concomitant with the gene expression profile indicating a more mature muscle tissue, myotube diameter also increased over time, and after 5 weeks of differentiation, skeletal muscle tissues were composed of tridimensionally distributed myotubes that displayed sarcomeric organization and sarcomeric lengths similar to human skeletal muscle fibers in vivo (Cutts, 1988). Overall, these data demonstrate that 3D skeletal muscles grown in the Biowire II platform can be maintained much longer than myotubes in standard 2D culture (which is typically not much longer than 10 days), and that these differentiated tissues display a gene expression profile and morphology more consistent with mature human skeletal muscle fibers.

The ability of skeletal muscle to contract following an electrical stimulus (e.g., an action potential from an α‐motor neuron) is a key physiological aspect that cannot be fully recapitulated in standard 2D culture systems. For example, when cultured in 2D, we were able to maintain cells for up to 11 days, after which point the cultures began to progressively detach from the plate. As skeletal muscle cells can only be cultured for this short period in 2D, there is a limitation to the extent of maturation that can be achieved by time in culture. When compared to the Biowire skeletal muscle tissues, the 2D skeletal muscle cells had significantly reduced *MYH1* and *MYH2* expression, had fewer myotubes and myotubes were less aligned. Myotube number, length, size, alignment and orientation with respect to the axis of actuation are all known to be key contributors to force generation by skeletal muscle (Pagan‐Diaz et al., 2018). Additionally, an important hallmark of skeletal muscle is the ability to generate individual twitch contractions (evoked by a low frequency stimulus) as well as tetanus (evoked by high frequency stimuli) in which fusion of successive muscle twitch contractions results in a single sustained high force contraction. Our engineered skeletal muscle tissues displayed both twitch (in response to 1 Hz) and completely fused tetanus (in response to 100 Hz) following electrical stimulation on day 7 of differentiation. Moreover, tetanic forces increased in response to higher stimulation frequencies and to increasing maturation time, which in our case was day 35 of differentiation.

We then utilized our ability to electrically stimulate 3D skeletal muscle tissues to apply an “exercise” stimulus by chronically stimulating tissues over several weeks. Chronic stimulation resulted in enhanced myotube formation, as well as increased force production when compared to unstimulated tissues. Specifically, after 2 weeks of electrical stimulation, 3D skeletal muscle tissues generated twitch and tetanic forces 4‐fold and 8‐fold greater, respectively, than forces generated by time‐matched unstimulated tissues. Increased force in response to electrical or optogenetic stimulation, a genetic technique that stimulates cells through light, is consistent with previous observations in skeletal murine and human models (Huang et al., 2006; Ito et al., 2014; Khodabukus et al., 2019; Raman et al., 2016). However, our stimulated tissues generate tetanic forces up to 33 μN/mm^2^ at D21 and D28 (after 2 and 3 weeks of electrical stimulation, respectively), which are significantly higher than forces generated by previously reported primary human myoblast‐derived in vitro muscle models, recently reviewed by Vesga‐Castro and colleagues (Vesga‐Castro et al., 2022). Additionally, the tetanic (100 Hz)/twitch (1 Hz) force ratio of our exercised tissues, after 2‐week chronic stimulation, resulted in a ratio of 31.95 ± 11.53. To our knowledge, our protocol results in engineered exercised skeletal muscle tissues with the highest tetanic/twitch ratio, compared with other proposed engineered skeletal muscle tissues, recently reviewed (Vesga‐Castro et al., 2022). However, as mentioned by Vesga‐Castro and colleagues, differences in determining cross‐sectional area and technical limitations in this procedure should be considered (Vesga‐Castro et al., 2022).

In terms of both twitch and tetanic forces in our stimulated 3D skeletal muscle tissues, we observed that while forces increased rapidly (e.g., after only 1 week of stimulation), by week 4 of stimulation, force values began to decrease. The decrease in force after 4 weeks of stimulation could be ascribed to the hypercontraction of myotubes over time resulting in some myotube breakage, though further studies are needed to fully understand the underlying mechanism. Similar to the twitch amplitude (force) and the mathematically related rise slope (contraction rate), exercise increased the rise time (time‐to‐peak) and decreased the half relaxation time during tetanic contractions relative to unstimulated tissues. Exercise also resulted in tissues with enhanced fatigue resistance as compared to unstimulated tissues. While enhanced fatigue resistance with chronic stimulation has been demonstrated in rodent myocyte‐derived engineered skeletal muscles (Khodabukus et al., 2015), to our knowledge, this is the first evidence of human engineered 3D skeletal muscle tissues able to display improved fatigability comparable with what is expected in an in vivo setting. Interestingly, Khodabukus et al. (Khodabukus et al., 2019) detected a decrease in fatigue resistance following chronic stimulation of human engineered 3D muscle tissues, which may have been due to the stimulation paradigm, which activated hypertrophic signaling, but not metabolic adaptations. Differences might also be ascribed to improved tissue maturity, or the tissue age, cell culture conditions, and/or features intrinsic to the Biowire platform. Additionally, the fatigability assessment protocol itself may be a contributing factor, as we assessed fatigability using a different tetanic stimulus than the other human 3D skeletal models (Khodabukus et al., 2019). The ability to recapitulate muscle fatiguability in tissue culture may help to shed light onto the mechanisms underlying a variety of diseases characterized by pathological fatigability and may accelerate related drug screening. Interestingly, enhanced force and fatigue resistance were correlated with a type IIx to IIa myosin heavy chain expression shift, which has also been observed as an adaptation to exercise training in humans in vivo (Short et al., 2005).

One of the requirements of engineering 3D skeletal muscle systems is to obtain more predictive models for drug screening than those currently available, thereby improving the translation to clinic. To test the pharmacological predictivity of our model, we exposed 3D skeletal muscle tissues to dexamethasone, which has a well characterized mechanism of action in skeletal muscle. A two‐week exposure to dexamethasone resulted in a dramatic decrease (up to 90%) in both twitch and tetanic forces in engineered skeletal muscle tissues. Glucocorticoid‐induced weakness is widely attributed to increased protein breakdown and myofiber atrophy, driven by upregulated expression of proteolytic genes (Schakman et al., 2013; Shin et al., 2000; Stitt et al., 2004). In support of this mechanism driving impaired muscle contractility in our engineered skeletal muscle tissues, RNAseq analysis identified the atrogenes MAFbx and MuRF1 (FBXO32 and TRIM63, respectively) as being highly upregulated following dexamethasone treatment, along with a marked upregulation of the transcription factor FOXO1, which promotes transcription of these genes and apoptosis. Further investigations will address tissue atrophy as a reduction in myotube diameter, as previously reported in human (Khodabukus et al., 2020) and murine 3D skeletal models (Shimizu et al., 2011) treated with dexamethasone. Altogether, impaired muscle contractility and increased expression of atrophy‐associated genes following dexamethasone treatment demonstrate the utility of our skeletal muscle platform for testing the effects of pharmacological agents on skeletal muscle function. Thus, these data support the main application of the platform, which is for drug discovery, development, and screening for use by the pharmaceutical industry at scale.

In summary, 3D skeletal muscle tissues that were engineered using the Biowire II platform display morphological and contractile properties that closely resemble human skeletal muscle in vivo. Our engineered tissues also demonstrated gene expression, fatigability and contractile responses to “exercise” stimulation and a pharmacologic treatment that are consistent with known human muscle adaptations to these stimuli. This engineered human skeletal muscle tissue platform may offer many advantages over standard 2D cultures and can be used as a powerful tool for basic muscle biology research, as well as for testing the effects of therapeutic drugs on enhancing muscle function.

Future studies are needed to explore the full capabilities of this platform. For example, the experiments here were performed using primary myoblasts isolated from a young healthy donor, which may not be representative of the population that is intended to be treated. Future studies will utilize myoblasts isolated from patients with genetic muscle disease or iPSC‐derived myoblasts from diseased patients, which would provide a more relevant context for screening therapeutic drugs. Another way by which this platform may be adapted is to alter the chronic electrical stimulation protocol to generate tissues that are more representative of either fast‐twitch or slow‐twitch skeletal muscles. A myofiber type is determined by the α‐motor neuron that innervates it, and it may be possible to modulate the frequency at which the skeletal muscle tissues in this platform are stimulated to drive the tissue to adapt towards a more slow‐or fast‐twitch phenotype (Huang et al., 2006). This could provide a system for studying the mechanisms regulating myofiber type determination and could allow for drug screening on both fast‐and slow‐twitch muscle tissues, which would be relevant for muscle diseases in which specific fiber types are more affected. A logical progression of our platform will also consist of adding complexity to the system, by establishing neuromuscular junctions, aiming to identify novel therapeutic treatments for patients affected by neuromuscular diseases (Charoensook et al., 2017; Cvetkovic et al., 2017; Osaki et al., 2018; Vila OF et al., 2021).

## AUTHOR CONTRIBUTIONS

I.P. and M.J.S. contributed equally; I.P., M.J.S., R.A.S., N.B, K.M.D., T.S. and M.P.G., designed research; I.P., M.J.S., B.S., D.R.G., K.C., S.S., V.B., J.E.N., R.A.S., performed research; I.P., M.J.S., Q.S., R.A.S., N.B., K.M.D., T.S. and M.P.G. analyzed data; I.P. and M.J.S. wrote the manuscript; T.S. and N.T.F. reviewed the manuscript.

## FUNDING INFORMATION

The authors received no financial support for the research, authorship and for the publication of this article.

## CONFLICT OF INTEREST STATEMENT

I.P., B.S., K.C., V.B., J.E.N., S.S., M.G., N.T.F., K.M.D., R.A‐S., N.B., M.P.G are employees and shareholders of Valo Health. M.J.S., D.R.G., Q.S., and T.S. are employees and shareholders of Regeneron Pharmaceuticals.

## ETHICS STATEMENT

Primary human myoblasts used in these studies were obtained commercially from Cook MyoSite, Inc., and thus, these studies were exempt from IRB approval.

## Supporting information


**Figure S1.** Skeletal muscle cells differentiated in 2D for 10 days had increased mRNA expression of MYOG, MYH1, and MYH2, as compared to Day 0 of differentiation (Ai). Unstimulated Biowire tissues cultured for 21 days expressed more MYH1 and MYH2 than 2D skeletal muscle cells (Aii). Bars represent mean ± SEM (*n* = 2). Representative bright‐field images: skeletal cells differentiated in 2D for 10 days have myotubes with random orientations (Bi), whereas Biowire skeletal tissue has aligned myotubes that are oriented along the axis of actuation (Bii).

**Figure S2.** Electrical stimulation (exercise) results in skeletal muscle tissues with improved force. Representative traces of contractility of 2‐week‐stimulated and time‐matched unstimulated skeletal muscle tissues, showing higher forces generated by stimulated tissues at each stimulation frequency.

**Figure S3.** Both unstimulated and stimulated tissues express MYH7 starting from D21.

**Figure S4.** Chronic exposure to dexamethasone modulates the kinetic parameters of skeletal muscle tissues. One‐ and two‐week dexamethasone treatment increases decay time (half‐time relaxation) in stimulated tissues as compared to unstimulated tissues (asterisk on the bracket). Additionally, 1‐week dexamethasone treatment increases decay time in stimulated tissues, as compared to vehicle control (asterisk above the bar), while 2‐week dexamethasone treatment significantly decreases decay time in unstimulated tissues, as compared to vehicle control (asterisk above the bar) (Ai). However, dexamethasone treatment does not affect rise time (time‐to‐peak) in either stimulated or unstimulated tissues (Aii). Data presented as mean ± SEM (*n* = 8 tissues, two technical replicates) *p < 0.05; **p < 0.005; ***p < 0.0005; ****p < 0.0001.

**Movie S1.** Myotubes are tri‐dimensionally distributed in skeletal muscle tissues. Projection of a representative immunofluorescence image of skeletal muscle tissues composed of several myofibers distributed in a 54 mm z stack, stained with fluorophore‐conjugated phalloidin to visualize F‐actin (green). Nuclei were stained with DAPI (blue).

**Movie S2.** Super‐resolution image of myotubes allows for visualization of sarcomeric structures. Projection of a representative myotube composing skeletal muscle tissues, stained with fluorophore‐conjugated phalloidin to detect F‐actin and acquired by a SoRa spinning disk confocal system (see Material and Methods).

**Movie S3.** Representative time‐lapse movies of skeletal muscle tissues resuspended in the Biowire II platform, acquired in brightfield at 2× magnification, showing twitch contractility, when stimulating at 1 Hz.

**Movie S4.** Representative time‐lapse movies of skeletal muscle tissues resuspended in the Biowire II platform, acquired in brightfield at 2× magnification, showing tetanic contractility, when stimulating at 100 Hz.

**Movie S5.** Representative time‐lapse movies of skeletal muscle tissues, acquired under UV light at 10× magnification, showing wire deflection during twitch contractility, when stimulating at 1 Hz.

**Movie S6.** Representative time‐lapse movies of skeletal muscle tissues, acquired under UV light at 10× magnification, showing wire deflection during tetanic contractility, when stimulating at 100 Hz.

## Data Availability

The data that support the findings of this study are available from the corresponding authors upon request. Source data from RNAseq analyses will be deposited in the Gene Expression Omnibus data repository upon publication.
