# Supplementary figures and images for: Electrical stimulation of biofidelic engineered muscle enhances myotube size, force, fatigue resistance, and induces a fast‐to‐slow‐phenotype shift
Source: Physiol Rep. 2024 Oct 9;12(19):e70051. doi: 10.14814/phy2.70051 (PMC11464147; doi:10.14814/phy2.70051)

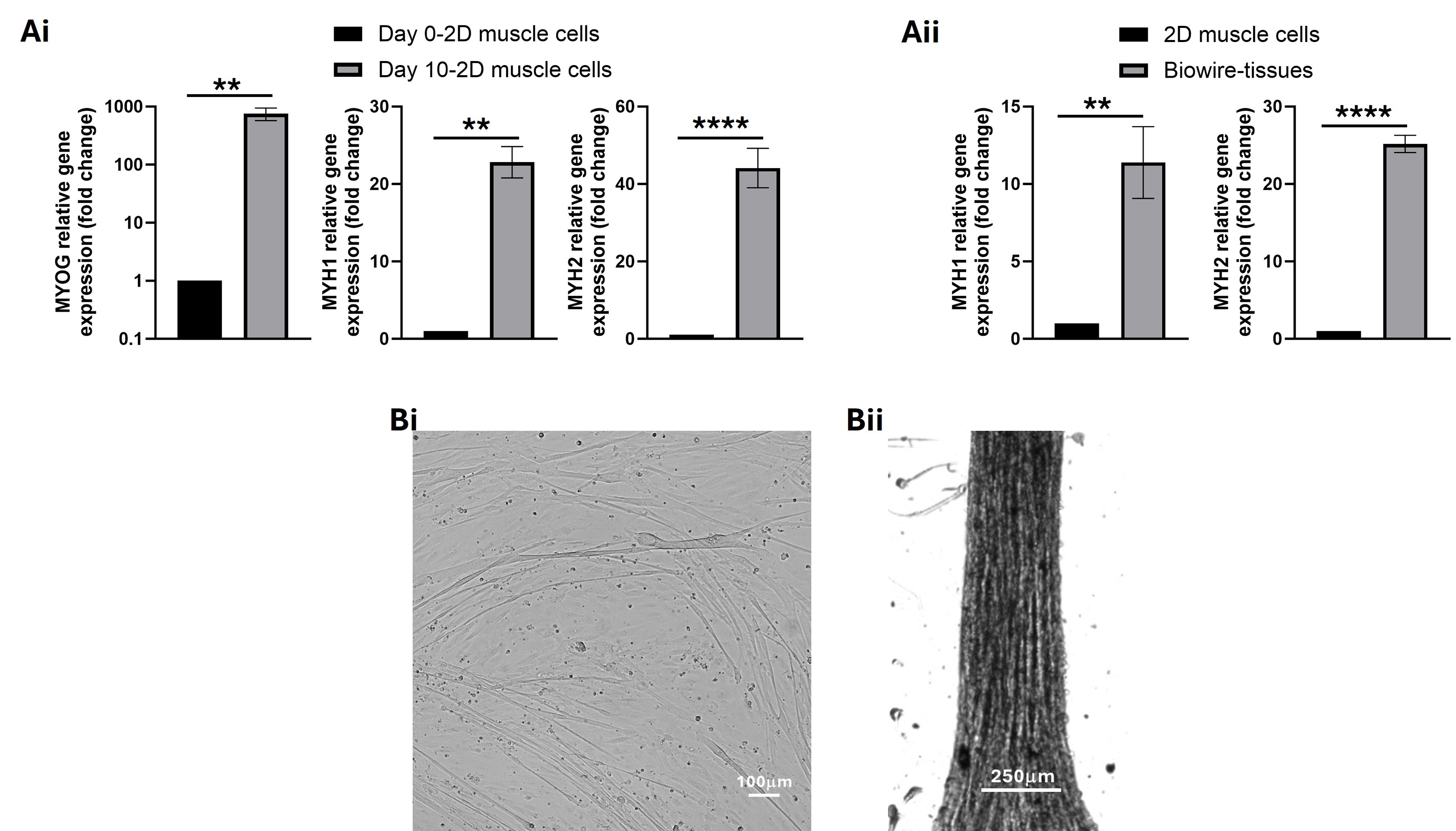

Supplement: Supplementary file 1 — Figure S1. Skeletal muscle cells differentiated in 2D for 10 days had increased mRNA expression of MYOG, MYH1, and MYH2, as compared to Day 0 of differentiation (Ai). Unstimulated Biowire tissues cultured for 21 days expressed more MYH1 and MYH2 than 2D skeletal muscle cells (Aii). Bars represent mean ± SEM (n = 2). Representative bright‐field images: skeletal cells differentiated in 2D for 10 days have myotubes with random orientations (Bi), whereas Biowire skeletal tissue has aligned myotubes that are oriented along the axis of actuation (Bii). [file PHY2-12-e70051-s004.jpg]

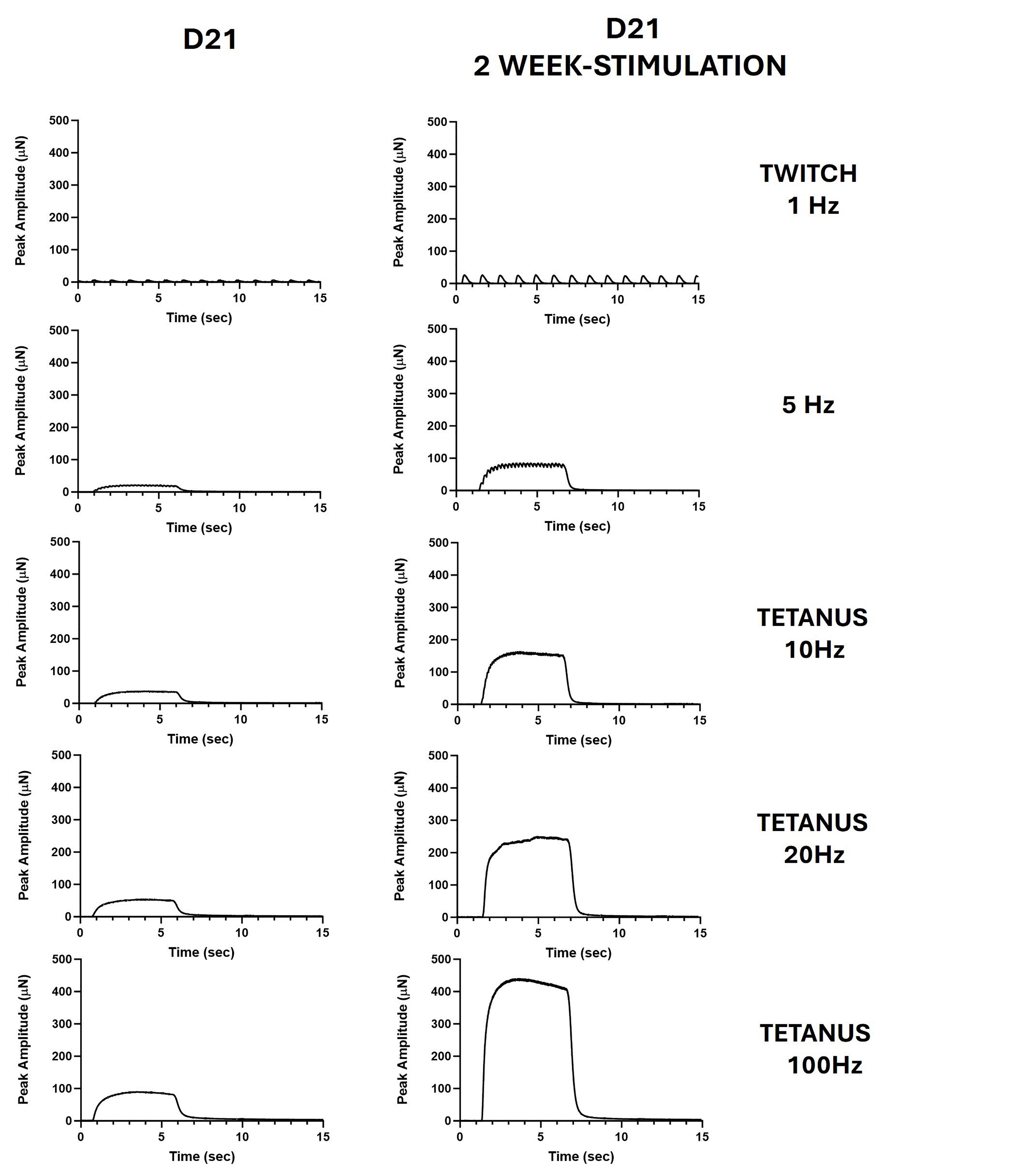

Supplement: Supplementary file 2 — Figure S2. Electrical stimulation (exercise) results in skeletal muscle tissues with improved force. Representative traces of contractility of 2‐week‐stimulated and time‐matched unstimulated skeletal muscle tissues, showing higher forces generated by stimulated tissues at each stimulation frequency. [file PHY2-12-e70051-s001.jpg]

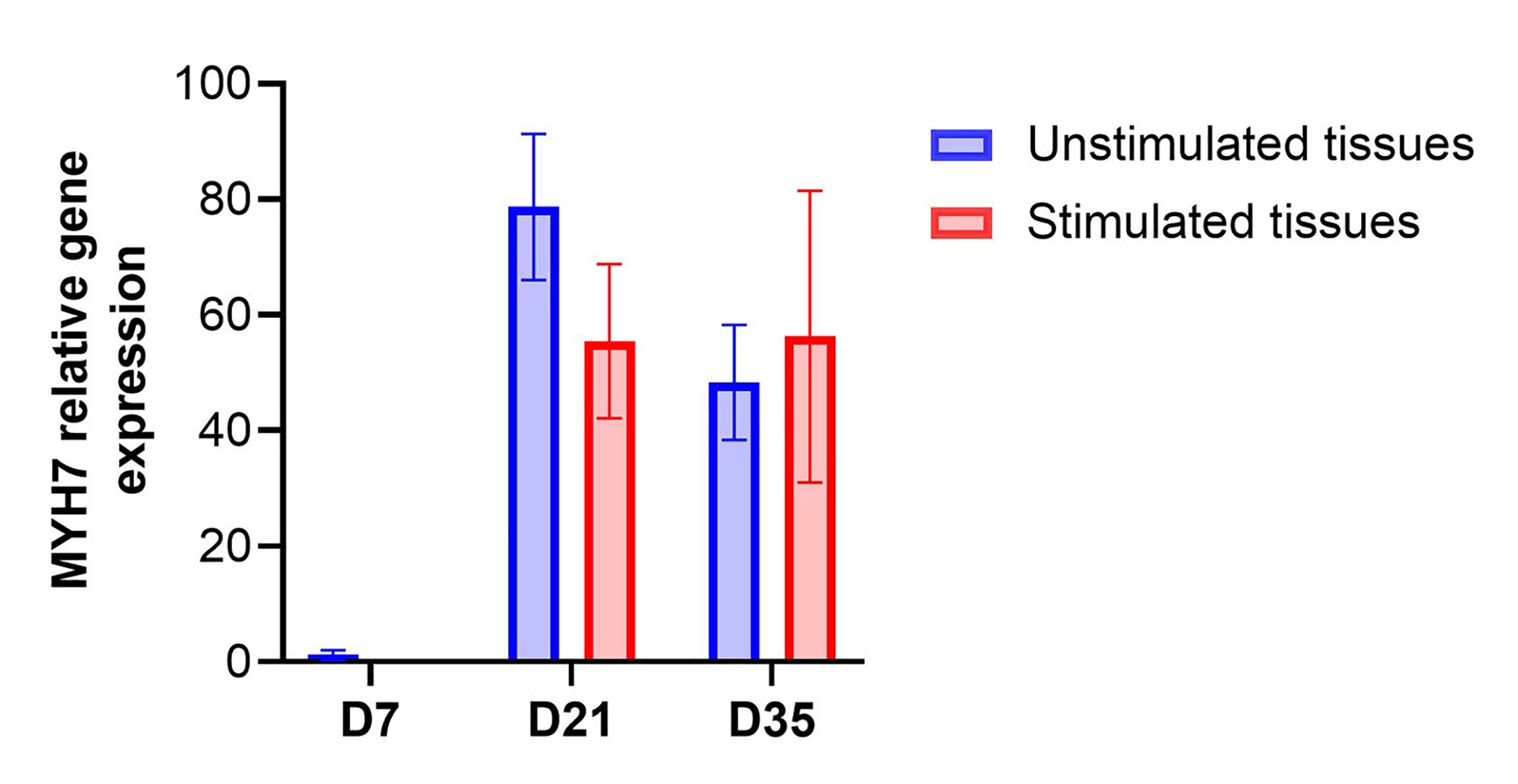

Supplement: Supplementary file 3 — Figure S3. Both unstimulated and stimulated tissues express MYH7 starting from D21. [file PHY2-12-e70051-s006.jpg]

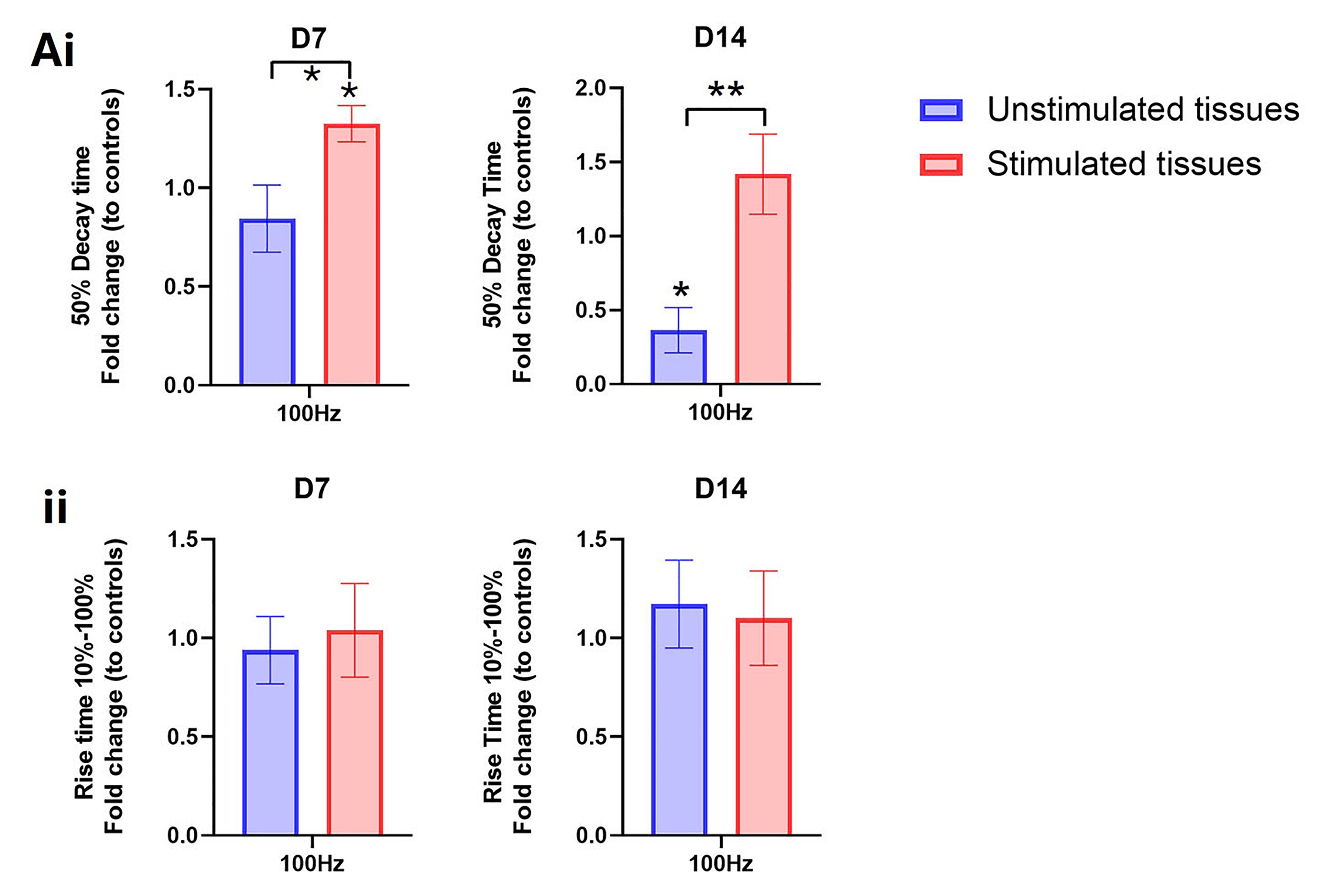

Supplement: Supplementary file 4 — Figure S4. Chronic exposure to dexamethasone modulates the kinetic parameters of skeletal muscle tissues. One‐ and two‐week dexamethasone treatment increases decay time (half‐time relaxation) in stimulated tissues as compared to unstimulated tissues (asterisk on the bracket). Additionally, 1‐week dexamethasone treatment increases decay time in stimulated tissues, as compared to vehicle control (asterisk above the bar), while 2‐week dexamethasone treatment significantly decreases decay time in unstimulated tissues, as compared to vehicle control (asterisk above the bar) (Ai). However, dexamethasone treatment does not affect rise time (time‐to‐peak) in either stimulated or unstimulated tissues (Aii). Data presented as mean ± SEM (n = 8 tissues, two technical replicates) *p < 0.05; **p < 0.005; ***p < 0.0005; ****p < 0.0001. [file PHY2-12-e70051-s002.jpg]
